# Electrical Parameters as Diagnostics of Fresh Engine Oil Condition—Correlation with Test Voltage Frequency

**DOI:** 10.3390/s23083981

**Published:** 2023-04-14

**Authors:** Artur Wolak, Ryszard Żywica, Jarosław Molenda, Joanna Katarzyna Banach

**Affiliations:** 1 Department of Quality and Safety of Industrial Products, Cracow University of Economics, Sienkiewicza 4 St., 30-033 Kraków, Poland; 2 Institute of Management and Quality, Faculty of Economics, University of Warmia and Mazury in Olsztyn, 10-719 Olsztyn, Poland; 3 Łukasiewicz Research Network—Institute for Sustainable Technologies, 6/10 Pułaskiego St., 26-600 Radom, Poland

**Keywords:** electrical parameters, voltage frequency, impedance magnitude, phase shift angle, conductance, susceptance, capacitance, quality factor, engine oil, total acid number, total base number

## Abstract

The aim of this study was to assess whether electrical parameters (capacitance and conductivity) of fresh engine oils—tested over a wide range of measurement voltage frequencies—can be used for oil quality assessment and its identification, based on physicochemical properties. The study encompassed 41 commercial engine oils with different quality ratings (American Petroleum Institute (API) and European Automobile Manufacturers’ Association (ACEA)). As part of the study, the oils were tested for their total base number (TBN) and total acid number (TAN), as well as their electrical parameters, including impedance magnitude, phase shift angle, conductance, susceptance, capacitance and quality factor. Next, the results for all of the samples were examined for correlations between the mean electrical parameters and the test voltage frequency. A statistical analysis (k-means and agglomerative hierarchical clustering) was applied to group oils with similar readings, drawing on the values for all electrical parameters to produce group oils with the highest similarity to each other into clusters. The results show that the electrical-based diagnostics of fresh engine oils can serve as a highly selective method for identifying oil quality, offering much higher resolution than assessments based on the TBN or the TAN. This is further supported by the cluster analysis, with five clusters generated for electrical parameters of the oils, compared to only three generated for TAN- and TBN-based measurements. Out of all the tested electrical parameters, capacitance, impedance magnitude and quality factor were found to be the most promising for diagnostic purposes. The value of electrical parameters of fresh engine oils is mostly dependent on the test voltage frequency (with the exception of capacitance). The correlations identified in the course of the study can be used to select for those frequency ranges that offer the highest diagnostic utility.

## 1. Introduction

Engine oils are manufactured by combining exact proportions of base oil with oil additives (including lubricants, alkaline additives, viscosifiers, antioxidants, detergents and other substances) in order to ensure that the oil provides sufficient lubricity, heat removal capacity, sealing, contaminant control and anti-corrosion protection [1]. With this gamut of functions, engine oil is seen a structural component of engines and a major determinant of their reliability/maintainability. Oil also happens to be the fastest-degrading element and thus the most frequently replaced. Lubricating oil undergoes gradual degradation brought by exposure to high temperatures, water, air and acidic contaminants (products of oil oxidization) [2]. The precise monitoring of oil deterioration is essential for both technical and economic reasons. Due to the severe and variable operating parameters of vehicles, engine oil aging has to be analyzed individually for each propulsion unit [3,4]. Most changes in lubricating oil parameters are difficult to detect in service. Similarly, it is difficult to detect when in-use oil reaches critical levels of its physical and chemical parameters [5]. Data on in-service lubricant oil condition is usually obtained by testing the selected parameters against fresh oil and/or comparing them against extreme values [4]. The precise measurement of engine oil degradation can be done via laboratory testing of sample batches extracted for analysis. Laboratory methods can be used to quantify a variety of oil parameters, including kinematic viscosity [6]; acid and alkaline index [7,8]; degree of oxidation, nitration and sulfonation [9,10]; water and glycol contents [11,12]; levels of wear elements (debris from friction pairs) [13], or antioxidant content [14,15]. Together, these readings can provide a full picture of oil quality. Conventional physicochemical testing methods used to monitor the quality and safety of engine oils tend to be time-consuming, costly, destructive and require multiple samples [16]. Therefore, new methods are needed for the quick assessment and control of oil quality [17]. A key factor in vehicle and machine operation is the timeliness of decision-making regarding working fluid quality [18]. Such decisions are made on the basis of on-the-fly measurements at the running site, without the need for complex set-ups and skilled analysts. There is therefore a need to develop and universalize equipment that can satisfy these criteria [19,20]. Electrical methods in particular offer a number of benefits vis-a-vis such applications, including quick measurement, low cost and high testing efficiency. In addition, the compact nature of electrical measurement equipment means that it can be used for field testing [21]. Electrical testing can be a valuable and easy-to-use tool for controlling engine oil quality as a result of its high reliability, sensitivity, short response (reaction) time and relatively low equipment costs [22]. Without the need to wait for laboratory results, diagnostic decisions on whether to extend or shorten oil change intervals can be made immediately. This can reduce the maintenance and running costs and even prevent serious failures [23,24].

Electrical properties are among the least understood physicochemical properties of engineering materials [22]. This is mainly due to gaps in our physics, biology, biophysics, technology and engineering knowledge. Further compounding the issue is the lack of appropriate testing equipment in general and specialized measurement sensors (especially dielectric sensors) in particular. Nevertheless, there are a few research centers around that world that have tackled this particular area of interest. Wu et al. proposed a novel type of inductive debris sensor consisting of two excitation coils and two sensing coils for online debris monitoring [25,26]. Similarly, Wang et al. focused on engine oil debris monitoring [27]. In order to boost the sensitivity of the inductance sensor, they utilized the mutual inductance of coils and the strong magnetic conductivity of permalloy to design a high sensitivity inductance sensor for the detection of debris in lubricating oil. A high sensitivity wear debris was also examined by Shi et al., Zhe et al. and Fan et al. [28,29,30]. A different approach was proposed by Heredia-Cancino et al. [31], who developed a sensor based on the position of the localized surface plasmon resonance (LSPR) of a band of silver nanoparticles (AgNP) synthesized by laser-assisted photoreduction. This plasmonic sensor can detect changes in the permittivity of the oil caused by the modification of the chemical properties of the lubricant. It is also worth noting that Masud et al. [32] presented a contactless interrogation technique and system for capacitive sensors via electromagnetic coupling. The sensor unit was composed of the capacitive sensor connected to a tuning capacitor and a coil forming an LC resonant circuit. The experimental results foresee the possibility to apply the proposed technique to different types of capacitive sensors, fostering, among others, applications based on low-cost printed labels as sensor units. Therefore, the primary objective of this study was to assess whether the electrical parameters (capacitance and conductivity) of fresh engine oils—tested over a wide range of measurement voltage frequencies—can be used for oil quality assessment and its identification, based on physicochemical properties. Learning and determining these relationships in fresh oils is a necessary step towards exploring the same for used oils. A key step towards achieving this research objective was to identify the effect of test voltage frequency on selected electrical parameters, as well as to verify whether correlations between these parameters occur at different frequencies. This will help identify the optimal range or the optimal frequency of test voltage in future research works.

## 2. Materials and Methods

### 2.1. Materials

A total of 41 commercially-available synthetic motor oils were selected for testing, all sourced from the same distributor. The oils were primarily selected for their viscosity class (5W30). All of the products had a synthetic base declared by the manufacturers, though most were not explicitly classified as either group IV or V. The oils included European, American and local (Polish) brands. A listing by manufacturer is presented in Table 1.

It is important to note that oil parameters varied from manufacturer to manufacturer (specifically in terms of car manufacturer specifications and API/ACEA quality rating). Table 2 groups the samples according to quality class, as per manufacturer information.

Most of the oils were rated ACEA A3/B4 or A5/B5 or C2, C3 or C4. The C3-rated oils—used in modern passenger cars and light commercial vehicles (both with diesel and gasoline engines)—were the most numerous (22 samples).

### 2.2. Electrical Testing Methodology

Electrical parameters of the tested oils were measured across a frequency range of 100 Hz to 1.2 MHz and an alternating voltage 200 mV. The parameters were measured using a precision LCR meter type E4980A (Agilent, Colorado Springs, CO, USA) and a measuring sensor constructed at the University of Warmia and Mazury in Olsztyn—the only scientific center in Poland (and one of the few in the world) that studies the applicability of electrical properties for the assessment of the quality and authenticity of technical materials (mainly fluids). Prior to the testing, supply line compensation was added, the measuring cell was electrically profiled and the test frequencies were chosen across a range of 100 to 1.2 MHz. Compensation of the cables (length 1 m) connecting the LCR meter to the measuring cell was carried out using the meter’s internal function. The capacitance of the cell was calculated from its physical dimensions (L × W × H: 94 × 24.5 × 78 mm) at approx. 2.6 pF, along with the required sample size of approx. 180 cm^3^. The sample temperature was approximately 20 °C. After a few series of preliminary measurements and accounting for the measurement precision of the E4980A meter, the following test frequencies were selected on the basis of previous testing: 100 Hz, 200 Hz, 300 Hz, 400 Hz, 500 Hz, 1 kHz, 2 kHz, 3 kHz, 4 kHz, 5 kHz, 10 kHz, 15 kHz, 100 kHz, 500 kHz and 1.2 MHz. The electrical parameters tested were impedance magnitude (*|Z|*), phase shift angle (*δ*), conductance (*G*), susceptance (*B*), parallel equivalent capacitance (*Cp*) and quality factor (*Q-factor*). The selected electrical parameters of 41 engine oils shows in the form of frequency characteristics. The values represent the means of two readings with a difference of no more than 1%. Other data were discarded as unreliable. In order to avoid electrolysis and polarization phenomena at the oil-metal interface, electrical parameters were measured above the frequency of 100 Hz. The effect of the possible occurrence of the “double-layer” phenomenon at the electrode-material interface (engine oils) error in comparative studies were omitted without prejudice to the achievement of the research aim.

**Figure 1:**
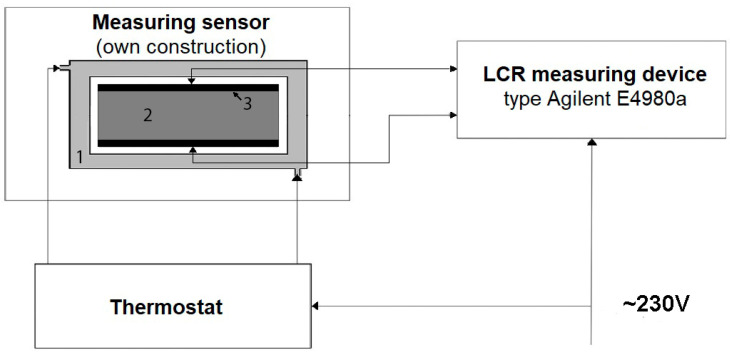
Diagram of the measurement system for testing electrical properties of engine oil. (1)—water jacket; (2)—sample; (3)—glass container with electrodes (measuring cell).

Engine oil was poured into glass containers (measuring cell) with two plate electrodes mounted inside. The electrodes were made of acid-proof steel and were tightly fitted to the opposite (larger) walls of the container. Afterwards, the samples were transferred into a Memmert ICP 500 climatic chamber (GmbH + Co. KG D-91126, Schwabach FRG, Germany). Once the tested oil had reached the target temperature (20 ± 0.1 °C), the containers with the samples were transferred to a metal container fitted with a water jacket and connected to a thermostat (PolyScience, Niles, Illinois, USA), to prevent uncontrolled temperature changes of the test subject oil. Next, the electrical conductance and capacitance parameters were measured in the measurement system presented in Figure 1.

### 2.3. Physicochemical Testing Methodology

Total base number (TBN) and total acid number (TAN) were chosen as the test parameters, the rationale being that the electrical parameters are influenced by polar additives that increase the alkali reserve (TBN), as well as by oil degradation products, whose levels can be detected as the TAN. Viscosity parameters were not included in the study, since viscosifiers tend to be polymers; for example, polyalkyl methacrylate, polyisobutylene and olefin copolymers with molecular weights typically larger than 10,000 g/mol (and that thus have only a small dipole moment) [33]. The choice was also dictated by the fact that the tested oils were fresh; in spent oil, viscosity is also affected by high-molecular-mass degradation products, making the inclusion of viscosity parameters more justified.

TBN was measured according ASTM D 2896 [34]. The total base number determination was performed by titrating a solution of a weighed quantity of the tested substance in a solvent (which contained an indicator). A solution containing toluene, isopropyl alcohol and water was used as the solvent. Titration was performed with a standardized alcohol solution of hydrochloric acid (HCl). Potentiometric titration was used for the glass and reference electrode. The equivalence point was set by means of potentiometric reading of the buffer solution in the target potential (expressed in mV).

Total acid number (TAN) was determined by titrating a solution of a weighed amount of the test substance in the solvent (containing an indicator). A solution containing toluene, isopropyl alcohol and water was used as the solvent. The acid number was then determined by potentiometric titration [11]. Titration was done using a standard alcoholic potassium hydroxide solution. TAN was determined in accordance with ASTM D 664 [35].

### 2.4. Statistical Analysis

The first step of the statistical analysis was to plot the electrical parameters as a function of the measurement frequency. Average values, standard deviations, min/max values, the coefficient of variation, median and the range of 25% to 75% are given in table and graph form. The coefficient of variation is the quotient (%) of the absolute variation value and the average value for the parameter. The coefficient was calculated by the traditional method and used to assess the degree of variation across the tested samples (by quantifying the strength of the variable), as well as to calculate the arithmetic mean. A high value of the coefficient corresponds to strong variation and, by the same token, a low value indicates weak variation. The purpose of this step was to plot the variability for all the oils, i.e., which parameters were highly variable and to what extent. Such data will be particularly useful for comparing fresh oil with spent oil—a subject for further research. On the one hand, if we assume that spent oil should have high variability within the selected frequencies, then low variability would be considered desirable in fresh oil. On the other hand, a large variation for fresh oils is a valuable finding for identifying those fresh oil components that account for the noted differences (whether it is the oil base, additives, or others). This step does not yet provide a clear answer as to which parameter is the most representative, as the results have to be compared against those for spent oils.

The second step of the statistical analysis was to group oils with similar values. A cluster analysis (k-means) was used to this end. The data were standardized (variable standardization) beforehand, since the reading covered a large span of scales. Five clusters were constructed for each electrical parameter, grouping oils that are most similar to each other in terms of the values obtained across the measurement frequencies.

The third stage also called for a cluster analysis, this time using the dendrite method. This step was designed to group the tested oils based on the totality of the electrical parameters. The resultant clusters grouped oils most similar in terms of their general electrical properties. Ward’s method was used for clustering. The distance was measured using the Euclidean distance.

The analysis was performed with Microsoft Excel and Statistica 13.0 software. The correlation and multiple regression were calculated.

## 3. Results

### 3.1. Applicability of Electrical Parameters for Engine Oil Testing

The main part of the study was to assess the applicability of electrical parameters for engine oil identification and quality assessment and from that, for measuring deterioration. Engine oils are dielectrics by virtue of their chemical composition, but the admixture of various additives (including polar molecules) allows current to flow through the oil. Some types of oil are based on ester compounds, which also have a dipole moment. This means that, in some cases, the oil base itself may also account for some of the electrical properties. Thus, it was necessary to select for electrical parameters that could be used to clearly distinguish between the different oils while also ensuring high measurement precision. As most electrical parameters correlate with the frequency of the applied voltage, it was also necessary to identify the best frequency ranges for diagnosing motor oil quality. Thus, this part of the study verified the diagnostic potential of the following parameters: impedance magnitude, phase shift angle, conductance, susceptance, capacitance and quality factor.

#### Impedance Magnitude

**Figure 2:**
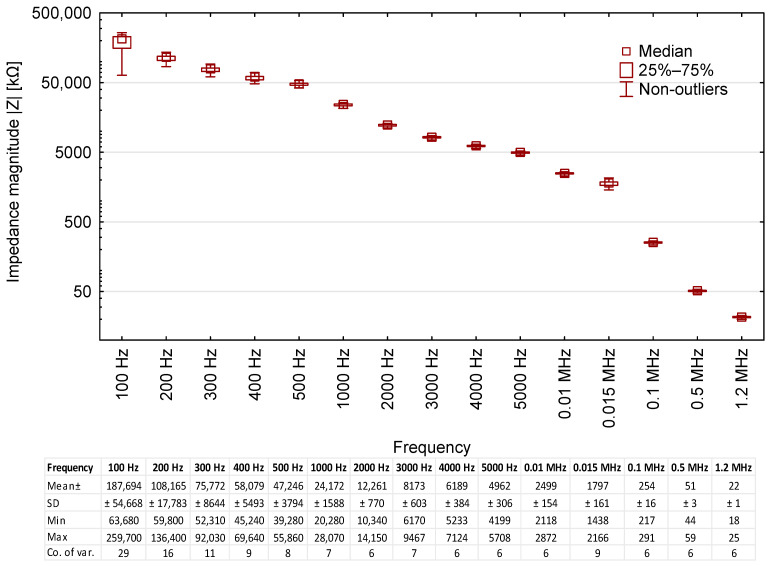
Impedance magnitude—|*Z*| readings for the tested oil samples, including base data for the results.

The correlation between *|Z|* and the frequency of measurement voltage is presented in Figure 2.

The impedance magnitude of fresh oil was found to decrease as the measurement frequency increased, starting from 259,700 kΩ at 100 Hz to 18 kΩ at 1.2 MHz. In other words, the higher the frequency of the measurement voltage, the better the electrical conductivity of the oil. There was also a noted decrease in the standard deviation and coefficient of variation, with the lowest value being 6% (from 4000 Hz onwards). The variance of the readings was inversely correlated with the measurement frequency, a trend particularly evident for 100 and 200 Hz. This means that low-frequency measurements can be useful for distinguishing between oils of different quality ratings (according to API or ACEA).

### 3.2. Phase Shift Angle

**Figure 3:**
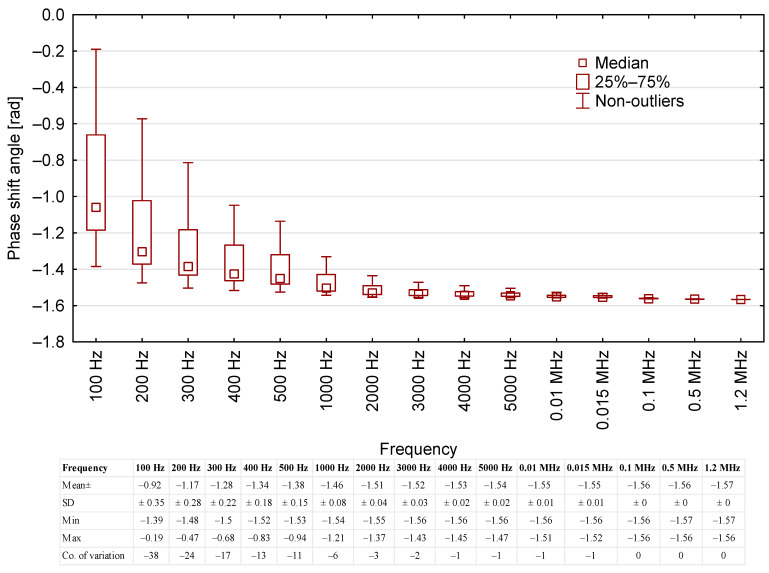
Correlation between phase shift angle readings for the tested oil samples and the test voltage frequency, including base data for the results.

The phase shift angle was also measured as a function of test voltage frequency. The resultant mean values are presented in Figure 3.

The phase shift angle was inversely correlated with the frequency. The highest variability of the phase shift angle (*δ*) was recorded at the frequency range of 100 Hz to 500 Hz, whereas the lowest variance was noted for frequencies of 4000+ Hz (coefficient of variation = 0 at frequencies higher than 0.1 MHz). Furthermore, the means for frequencies above 4 kHz had low standard deviation, no higher than 0.02. Notably, the readings for 0.1; 0.5; and 1.2 MHz were the same across all of the oil samples.

### 3.3. Conductance

**Figure 4:**
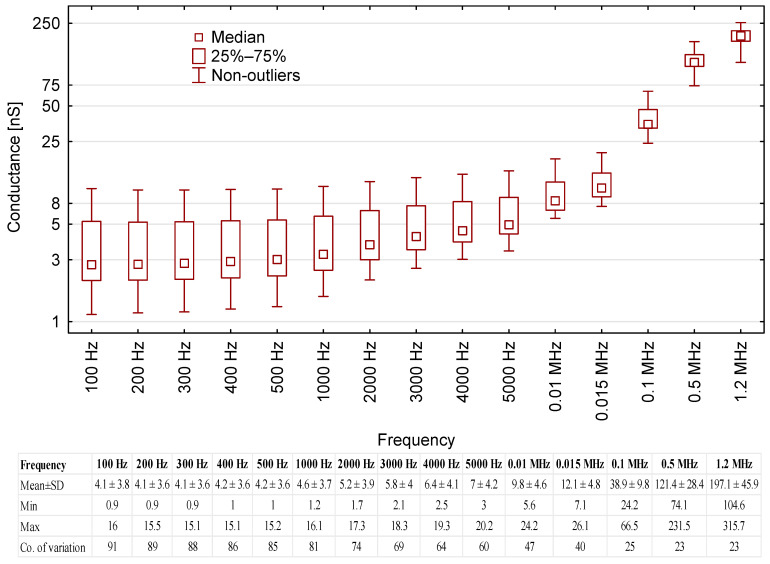
Correlation between conductance readings for the tested oil samples and test voltage frequency, including base data for the results.

Conductance was also among the parameters tested. The mean conductance of the tested motor oils as a function of test voltage frequency is shown in Figure 4.

The results show that conductance rose together with the voltage frequency, meaning that the oil samples became more conductive to electricity. Significant increases in conductance were noted from 0.01 MHz onward (the average conductance was 9.8 nS at 0.01 MHz and 197.1 nS at 1.2 MHz). However, the conductance readings exhibited high variability across all of the frequency range. The lowest coefficient variance value was 23% (at 0.5 MHz and 1.2 MHz), whereas peak variance (91%) was observed at 100 Hz. Thus, conductance was found to be of little use in oil engine quality assessment, authenticity verification and in-service quality monitoring.

### 3.4. Susceptance

**Figure 5:**
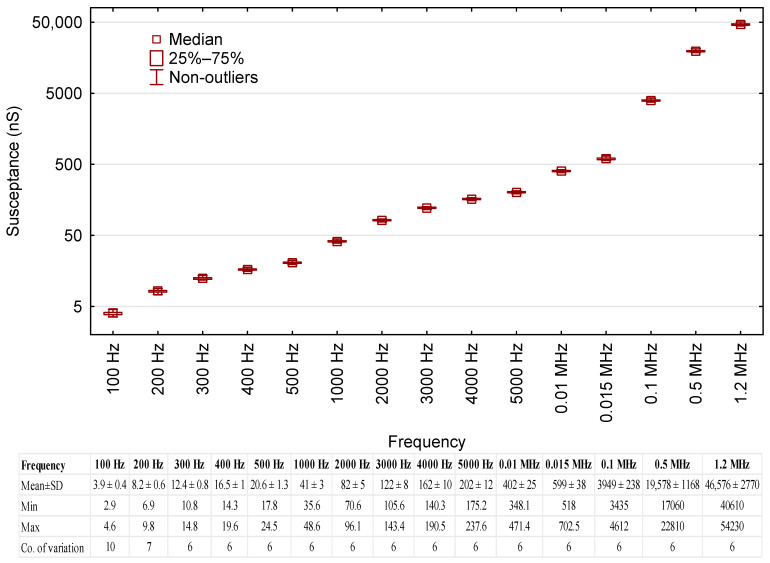
Susceptance readings for the tested oil samples, including base data for the results.

The mean susceptance values for the tested motor oils are collated in Figure 5.

An analysis of susceptance as a function of test voltage frequency shows that the two variables are positively correlated. Susceptance rose from 3.9 nS at 100 Hz to 46,577 nS at 1.2 MHz. Also of note is the high stability of the coefficient of variance, which was around 6% for most of the readings. The only higher values were recorded for 100 and 200 Hz (coefficient of variation = 10% and 7%, respectively). The assorted readings for the individual oils at different frequencies had low coefficients of variation, suggesting that susceptance can be a useful parameter for the in-service diagnosis of engine oil quality. However, comprehensive research on used oil is needed to fully confirm this finding.

### 3.5. Capacitance

**Figure 6:**
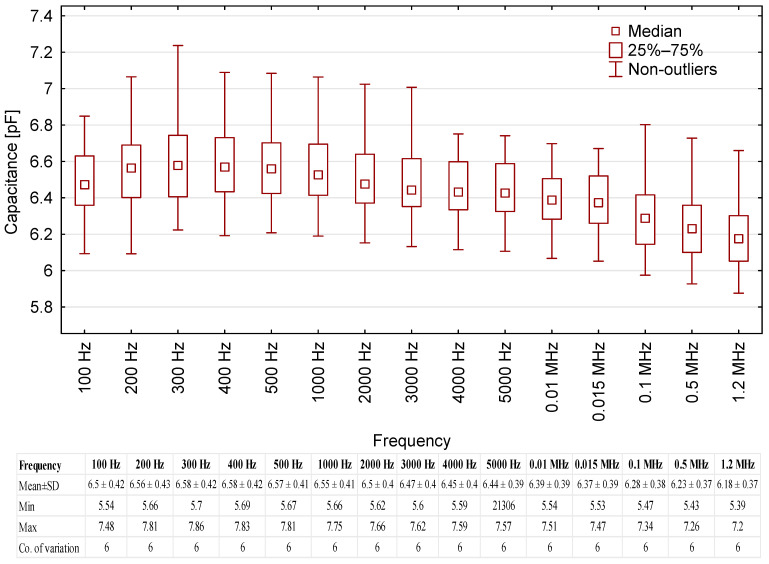
Capacitance readings for the tested oil samples, including base data for the results.

The capacitance of the motor oils in the test system was also measured. The correlation between mean capacitance and the frequency of test voltage is presented in Figure 6.

The results indicate that the capacitance of the fresh motor oil samples was similar across all voltage frequencies, with any differences falling within the margin of random error. Mean capacitance readings range between 6.18 to 6.58 pF. The readings are notably stable in terms of coefficient of variation, with low values of approx. 6% in all cases. Considering the quality characteristics of the results and their very low variation across the entire range of test voltage frequencies, this electrical parameter may prove the most suitable for the testing and monitoring of motor oils.

### 3.6. Quality Factor

**Figure 7:**
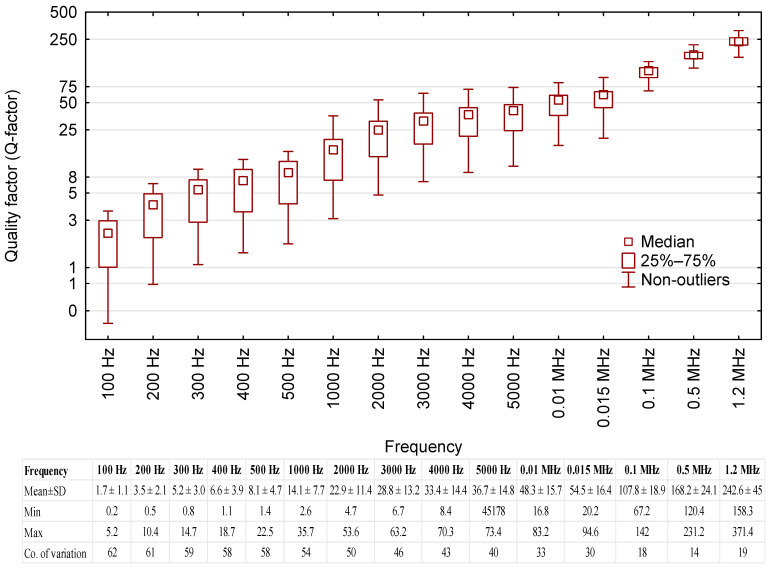
The quality factor of merit readings for the tested oil samples, including base data for the results.

The quality factor was also measured as a function of test voltage frequency. The results are shown in Figure 7.

The readings shown in Figure 7 indicate that higher values of test voltage frequency led to higher mean values of the quality factor (*Q-factor*) for the fresh oil samples. Furthermore, higher frequency values led to lower coefficients of variation (lower dispersion of results for the given series of measurements). The optimal voltage frequency for measurement purposes is predicated on the adopted coefficient. It stands to reason that frequencies higher than 2000 Hz may be successfully used to selectively identify oil samples from different clusters. In the test frequency series below 2000 Hz, the *Q-factor* readings span a wide range between the minimum and maximum values (50% + coefficient of variation), which may limit the practical use of low-frequency measurements.

In conclusion, the results obtained in this stage of the study pointed to impedance magnitude, phase shift angle, capacitance and quality factor as the most useful indicators of the quality for the oils tested. Reliable and selective testing of engine oil quality requires a well-calibrated test voltage frequency—one that provides adequate measurement resolution to classify the oil into its respective group of samples (cluster) while maintaining the measurement precision within the cluster. To ensure this, fresh oils should be tested at frequencies of 2000 Hz or higher. This hypothesis requires further verification by tests on individual groups of test samples that are classified into defined clusters.

### 3.7. Statistical Analysis

For the next step of statistical analysis, k-means clustering tools (five clusters and ten iterations) were used to identify oils with similar electrical parameters. 

**Figure 8:**
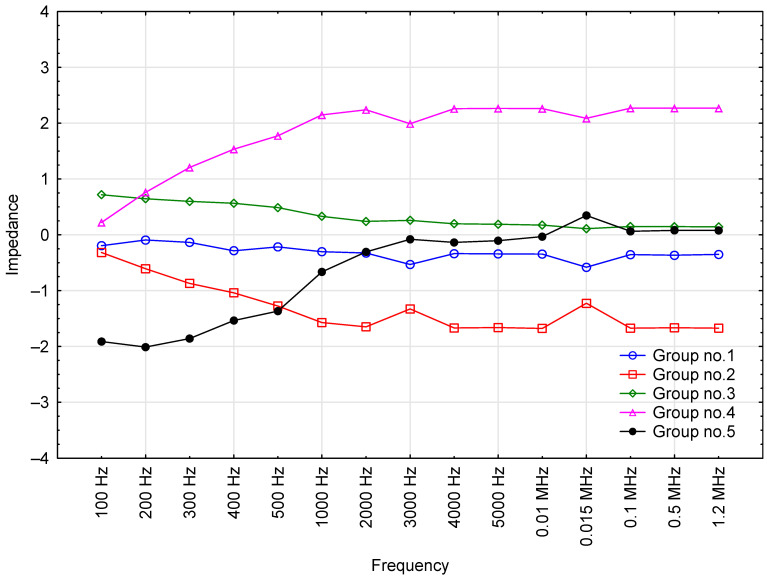
Effect of test voltage frequency on the average impedance magnitude of engine oils in each cluster (grouped by k-means).

The correlation between impedance magnitude and the frequency of test voltage across five clusters is presented in Figure 8. The assignment of the oils into the different groupings is presented in Table 3.

The impedance magnitude *(|Z|)* trends illustrated in Figure 8 indicate that the frequency range of 100 Hz to 2 kHz produced the highest increase in |*Z*| among Group 4 and 5 oils. In Group 4, *|Z|* values remained at around 2.2 (normalized value) above 2 kHz and were the most dissimilar to those in the other engine oil groups across the frequency range of 200 Hz to 1.2 MHz. These four outlying oils were: Fuchs_#1, Fuchs_#2, Millers_#3 and Millers_#4. A similar pattern, albeit much less pronounced, was observed for |*Z*|in Group 5, which comprised Elf_#3, Lotos, Mannol_#2, Mobil_#3 and Total_#2. The highest increases were observed within the frequency range of 100 Hz to 3 kHz. Frequencies of 100 to 300 Hz produced the lowest |*Z*| values in this group compared to the other oil groupings. Also notable was the near-complete disappearance of the differences between |*Z*| values of oils in Groups 3 and 5 at 3 + kHz, which means that there was no (significant) variation in oil |*Z*|. With the exception of *|Z|* values in Group 5 for 3 kHz and below, Groups 1, 3 and 5 should be considered very homogeneous (characterized by similar *|Z|* as a function of changes in the test voltage frequency).

**Figure 9:**
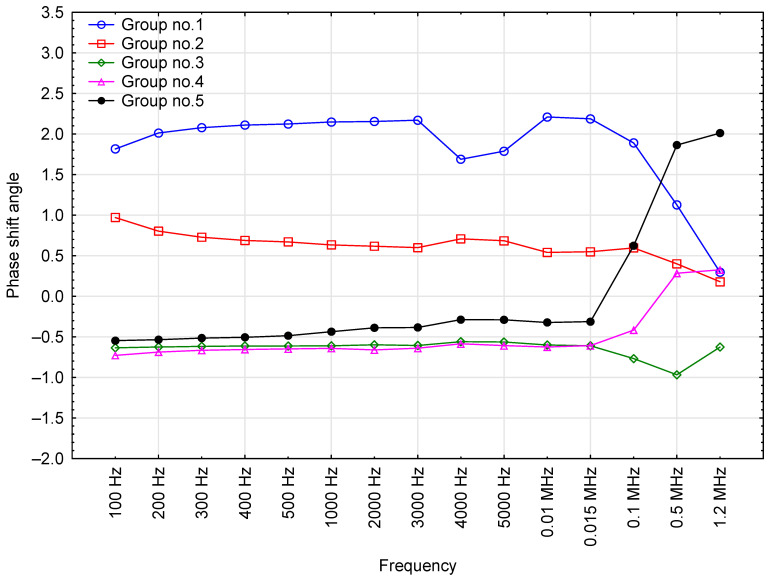
Effect of test voltage frequency on the average phase shift angle value for engine oils in each cluster (grouped by k-means).

The results obtained for the parameter ‘phase shift angle’ allowed for the distinguishing of two steps (Figure 9). The first encompassed the frequency range 100 Hz-15 kHz, while the second encompassed frequencies exceeding 15 kHz. For the first frequency range (step 1), oils (Elf_#3, Lotos, Mannol_#2, Mobil_#3, Total_#2) were included in Group 1, whereas oils (Castrol_#3, Eneos_#2, Eneos_#3, Eneos_#4, Mannol_#1, Millers_#3, Millers_#4, Revline, Total_#1) comprised Group 2 (Table 4). The oils of these groups exhibited higher values of the phase shift angle than the others. Groups 3, 4 and 5 were highly homogeneous in the frequency range of 100 Hz to 15 kHz. The second frequency range (above 15 kHz) marked a downward trend for oils that exhibited high phase shift angle values at lower frequencies, which is particularly evident in Group 1. The reverse was observed for Groups 4 and 5: low phase shift angle values at frequencies below 15 kHz, high values at frequencies above 15 kHz.

**Figure 10:**
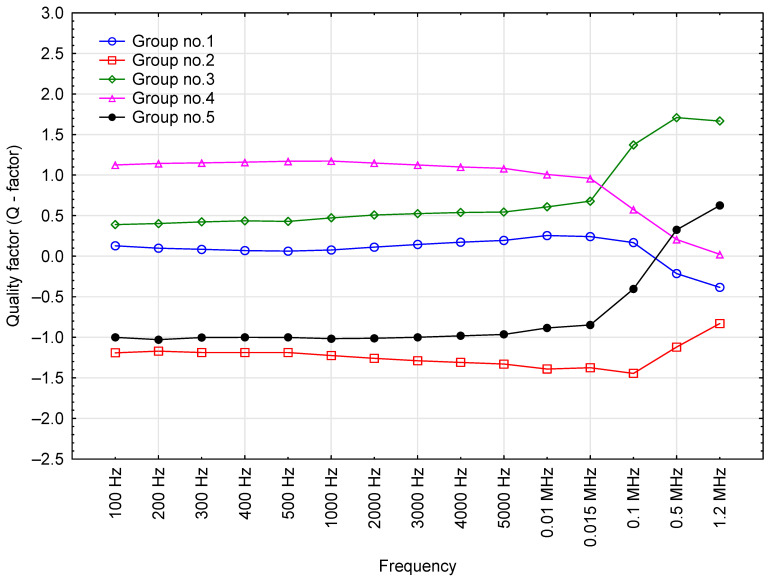
Effect of test voltage frequency on the average Q- factor value of engine oils in each cluster (grouped by k-means).

Oil grouping against the quality factor (*Q-factor*) was similar to the results for the phase shift angle (Figure 10). Again, the trend was reversed above the 15 kHz threshold. Oils that had higher values of the *Q-factor* at lower frequencies showed an increase in the value of this parameter and vice versa. There was high congruence in *Q-factor* values between Groups 2, 3 and 5, as well as between Groups 1 and 4 (Table 5).

**Figure 11:**
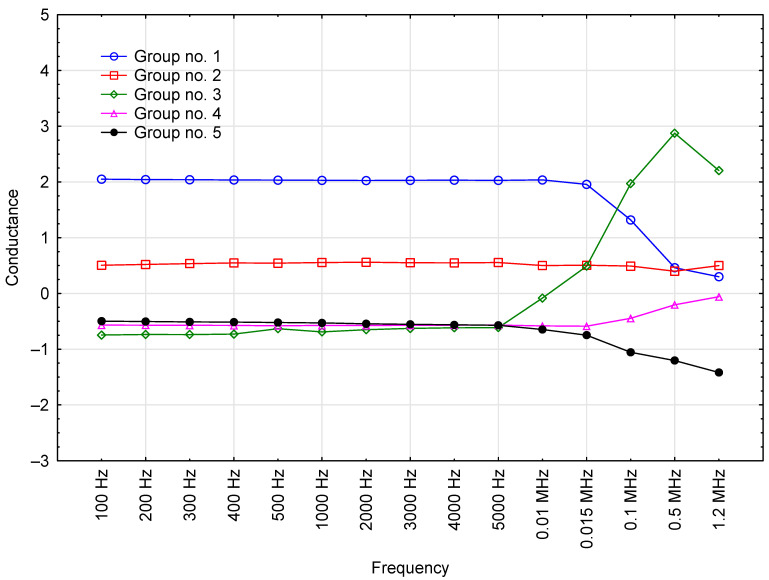
Effect of test voltage frequency on the average conductance value for engine oils in each cluster (grouped by k-means).

The groupings for conductance clearly demonstrate that oils Castrol_#3 and Elf_#2 (Group 3) are “sensitive” to test voltage frequency changes above 5 kHz, as illustrated in Figure 11. Conductance was virtually identical across Group 5 (Table 6) within the frequency range of 100 Hz to 5 kHz, but its value started to decrease above this range.

**Figure 12:**
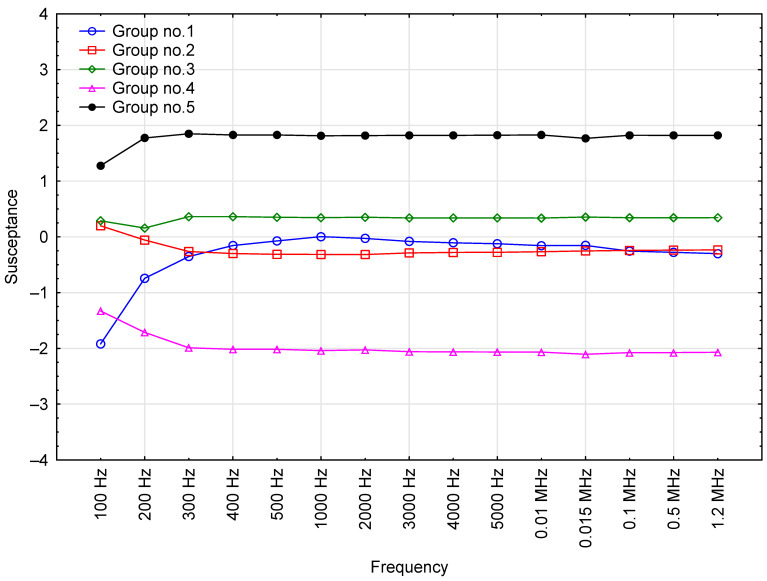
Effect of test voltage frequency on the average susceptance value of engine oils in each cluster (grouped by k-means).

The clustered susceptance readings exhibit very stable variation at frequencies above 300 Hz (Figure 12). There are, however, certain fluctuations below this threshold, e.g., higher frequencies produced increased susceptance in Groups 1 and 5, whereas the other three groups showed a decrease instead. An analysis of groupings above 300 Hz clearly shows that Groups 4 and 5 (Table 7) were the most diverse in terms of susceptance.

**Figure 13:**
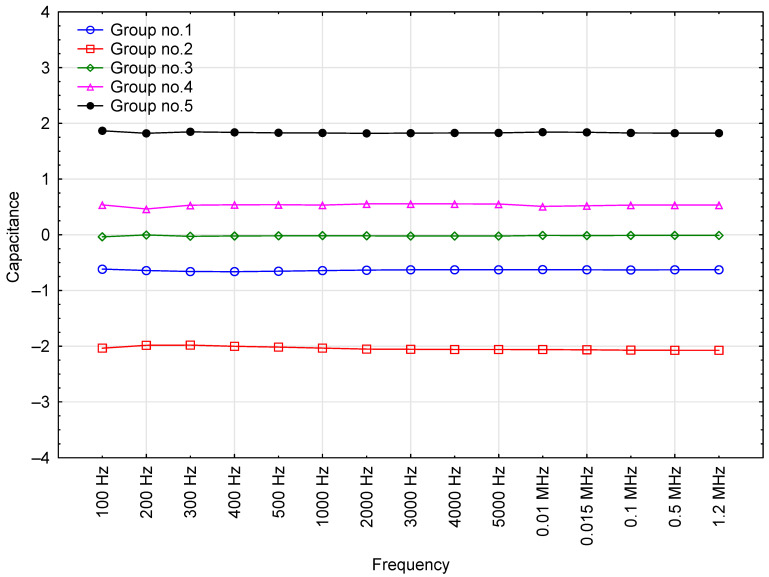
The effect of test voltage frequency on the average capacitance value of engine oils in each cluster (grouped by k-means).

These groupings show that capacitance was the only parameter that was stable over the entire frequency range (Figure 13). Moreover, there was clear variability of the capacitance values within engine oil groups, especially Groups 5 and 2 (Table 8). This makes it a very useful parameter for researchers developing methods for identifying fresh motor oils and testing purity over a wide range of test voltage frequencies.

It is also worth noting here that changes in frequency clearly result in completely different results for some samples. This can be seen in the conductance results for Castrol_#1 and Elf_#2; their conductance curve is stable at frequencies of 100 Hz-5 kHz, followed by a sudden spike at frequencies above 5 kHz.

To summarize the clustering results, Fuchs_#1, Fuchs_#2, Millers_#3 and Millers_#4 are crucially different from the other tested oils in terms of the electrical parameters measured. This difference is particularly pronounced for capacitance, susceptance and impedance magnitude. The results for conductance and phase shift angle at frequencies below 15 kHz are almost completely congruent, despite that fact that the oils are separated across two different groups. Only the *Q-factor* (quality factor) measurements fall outside this pattern of significant homogeneity across these four oils.

**Figure 14:**
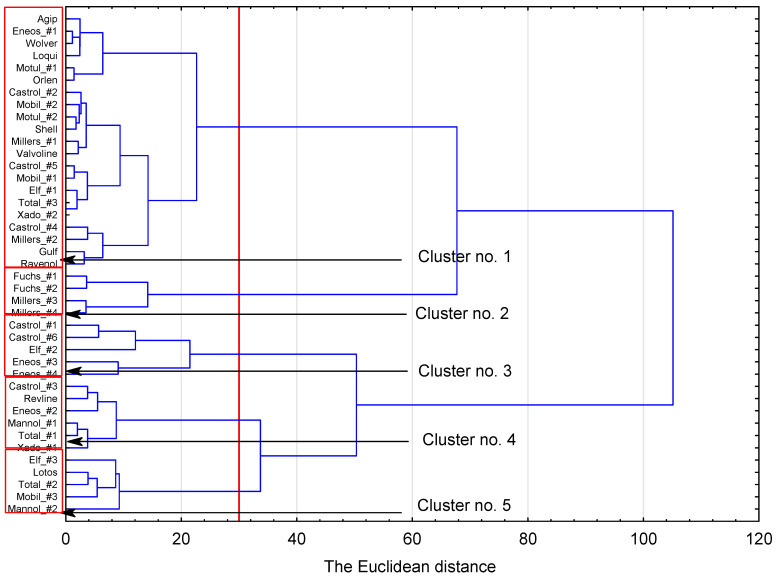
Agglomerative hierarchical clustering using Ward’s linkage and Euclidean distance - based on all of the electrical parameters combined.

**Figure A1:**
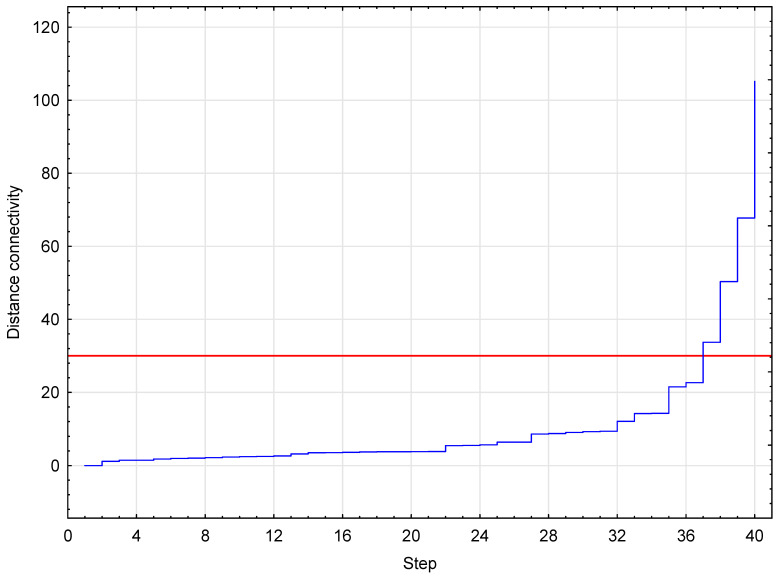
Clustering-over-time graph for the result presented in Figure 14: Agglomerative hierarchical clustering using Ward’s linkage and Euclidean distance. Blue line–Clustering-over-time graph, Red line–shows place where the clusters were split.

These findings are further supported by the dendrite clustering, which was used to select groupings based on all of the electrical parameters combined, rather than separately for each parameter, as in the previous analysis (Figure 14). The number of clusters was set based on the clustering-over-time graph (Appendix A, Figure A1), which shows the output of the clustering. The similarity measurements for the oils serve to demonstrate that Fuchs_#1, Fuchs_#2, Millers_#3 and Millers_#4 form a single cluster (Cluster No. 2) of the most highly similar oils. Castrol_#1, Castro_#6, Elf_#2, Eneos_#3 and Eneos_#4 were also found to be similar to each other and were grouped in Cluster No. 3. Cluster No. 4 comprises Castrol_#3, Revline, Eneos_#2, Mannol_#1, Total_#1 and Xado_#1. Cluster No. 5 groups Elf_#3, Lotos, Total_#2, Mobil_#3 and Mannol_#2 oils. Cluster No. 1 is the most numerous cluster, comprising 21 highly similar oils.

No correlations were found between the nominal quality rating of the oil (e.g., API) and its placement within the groupings. For example, oils Castrol#1 and Castrol#2 have a quality rating of SN, but were separated into two separate clusters by the dendrite algorithm (based on aggregated electrical parameters). Similarly, Eneos#2 and Eneos#4 are both rated SN/SM and CF by the manufacturer, yet ended up in different clusters based on the electrical readings. Therefore, a more comprehensive analysis is needed, one that would encompass all physicochemical parameters and thus help explain why, for example, samples of Eneos oils are scattered across three different groups or, by the same token, why the Elf and Castrol oils are in different clusters. This incongruity may stem from differences in quality ratings, oil bases, additive compositions (e.g., antioxidants, alkaline additives, or contaminant control/dispersing agents), or from other factors, such as the synergistic effects of lubricant ingredients. There is no doubt that synthesizing all electrical parameters for the clustering process might have affected its ultimate results, since the high coefficients of variation characteristic of conductance measurements may negatively affect the assignment of an oil into clusters. The next step of the study was to explain why specific oils were grouped as they were and, in particular, to answer the question of whether oils in one group have a common denominator in terms of quality, chemical composition, physicochemical parameters, etc.

**Figure 15:**
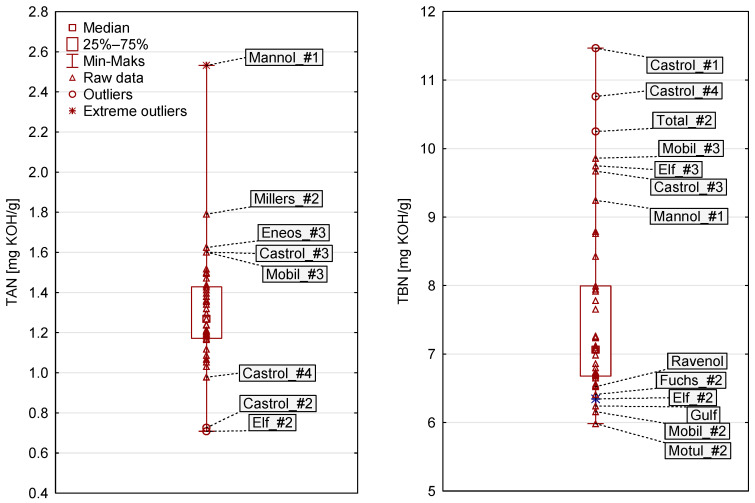
Engine oil TAN and TBN readings, including statistical assessment.

As part of this inquiry, an analysis of two chemical parameters (TAN and TBN) was conducted. The results obtained for fresh oils spanned a wide range, as detailed in Figure 15.

**Figure 16:**
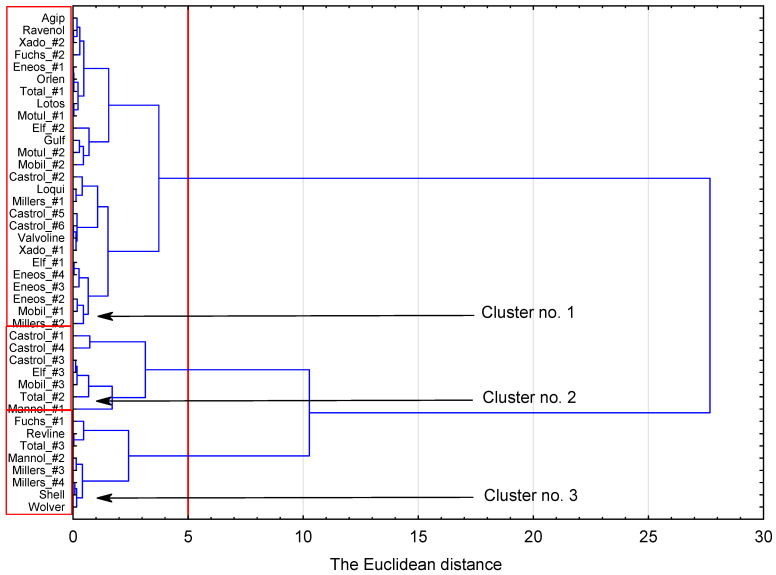
Agglomerative hierarchical clustering using Ward’s linkage and Euclidean distance - based on the TAN and TBN readings.

The next step of the study attempted to group the oils into clusters with maximum internal homogeneity and intergroup heterogeneity (based on the TAN and TBN readings). As before, an agglomerative cluster analysis was used. The results are presented in Figure 16.

**Figure A2:**
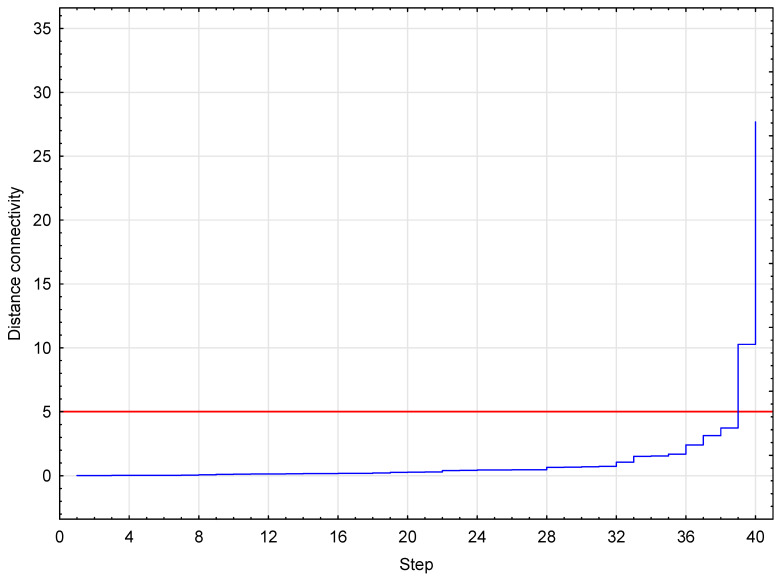
Clustering-over-time graph for the result presented in Figure 16: Agglomerative hierarchical clustering using Ward’s Linkage and Euclidean distance. Blue line–Clustering-over-time graph, Red line–shows place where the clusters were split.

The results indicate that fresh oils can be grouped into three clusters based on their TBN and TAN. The number of clusters was set based on the clustering-over-time graph (Appendix A, Figure A2). Cluster No. 3 includes Fuchs_#1, Millers_#3 and Millers_#4, which produced separate groupings in the electrical parameter clustering. This may suggest that there is a strong relationship between electrical parameters and TAN/TBN. Unfortunately, this finding was not entirely supported by the analysis, as Fuchs_#2, originally located near Fuchs_#1, Millers_#3 and Millers_#4, was assigned to a completely different grouping (Cluster No. 1). The analysis produced one more important finding by placing Clusters 2 and 3 at a large distance from Cluster 1, indicating that the oils are significantly dissimilar from each other (high heterogeneity between the groups).

## 4. Conclusions

The present study confirms that electrical-based diagnostics of fresh engine oils can serve as a highly selective method of identifying oil quality, offering a much higher resolution than assessments based on the alkali number or the acid number. This is further supported by the cluster analysis, as the classification based on electrical parameters produced five clusters of engine oils, whereas the TAN- and TBN-based measurements produced only three. The value of electrical parameters of fresh engine oils is mostly dependent on the test voltage frequency (with the exception of capacitance). The correlations identified in the course of the study can be used to select for those frequency ranges that provide consistent parameter values (regardless of voltage frequency), while ensuring the maximum selectivity for oils belonging to individual clusters.

It was found that, out of all the tested electrical parameters (impedance magnitude, phase shift angle, conductance, susceptance, capacitance and quality factor), capacitance, impedance magnitude and quality factor were the most promising for diagnostic purposes. Capacitance can be applied over the entire test voltage frequency range (i.e., from 100 Hz to 1.2 MHz), since the readings were independent of the frequency and featured low variability (coefficient of variation = 6%). The quality factor *(Q-factor*) provides useful readings within the frequency range of 2000 Hz to 0.01 MHz. Conversely, Q-factor measurements at frequencies below 2000 Hz were characterized by a high coefficient of variation, limiting their diagnostic utility. Impedance magnitude can be used in the frequency range of 4000 Hz to 0.01 MHz, exhibiting the lowest coefficients of variation above 4000 Hz and providing unambiguous selectivity for the oils in different clusters.

Electrical capacitance measurements show promise as a way to test the quality and authenticity of fresh engine oils across the entire range of the tested voltage frequencies. Impedance magnitude, phase shift angle and *Q-factor* also proved useful, but only within specific ranges. These findings are indicative of the capacitive-resistive and highly dielectric nature of the tested oils. This electrical measurement methodology is sensitive to changes in the chemical structure of engine oil components, even within the same API/ACEA quality rating. Thus, there is reason to look for strong relationships between the results of electrical measurements (which can be performed quickly) and the results of physicochemical parameter readings (which are time-consuming and require expensive equipment) and then to do the same for spent oils over a wide frequency range.

The results are also an essential step towards designing an electrical sensor, which, when installed in a car, would enable the owner to monitor engine oil quality and decide when to get an oil change. However, this also calls for further studies, ones which would include measurements for spent oil. Isolating and screening frequencies that could be used to clearly discriminate between fresh and spent oils is instrumental in opening the door for the development and deployment of such a sensor. In addition, establishing the full relationship between electrical and physicochemical parameters could be useful for creating an algorithm that could draw on such electrical readings to calculate the physicochemical parameters of engine oils.

## Figures and Tables

**Table 1:** List of tested oils by manufacturer.

Name of Manufacturer	Number of Oils	Name of Manufacturer	Number of Oils	Name of Manufacturer	Number of Oils
Castrol	6	Shell	1	Agip	1
Elf	3	Xado	2	Gulf	1
Eneos	4	Fuchs	2	Liqui Moly	1
Millers	4	Motul	2	Lotos	1
Mobil	3	Revline	1	Orlen	1
Total	3	Valvoline	1	Ravenol	1
Mannol	2	Wolver	1		

**Table 2:** Number of oils per each quality rating (ACEA, API).

**ACEA**				**Number of Oils**	**Oil Name(s)**
A *	B *		C *		
-	-		C3	22	Eneos_#1, Castrol_#5, Castrol_#6, Revline, Mannol_#1, Motul_#1, Elf_#1, Mobil_#1, Valvoline, Orlen, Fuchs_#2, Millers_#2, Millers_#4, Loqui, Ravenol, Gulf, Mannol_#2, Xado_#1, Agip, Lotos, Shell, Total_#3
A5	B5		-	4	Eneos_#3, Total_#2, Elf_#3, Mobil_#3
A3	B4		C3	3	Fuchs_#1, Wolver, Xado_#2
-	-		C2	3	Castrol_#2, Total_#1, Millers_#1
A1/A5	B1/B5		-	2	Castrol_#1, Castrol_#4
-	-		C2/C3	2	Millers_#3, Motul_#2
A1/A5	B1/B5		C2	1	Eneos_#4
A3	B3/B4		C3	1	Eneos_#2
A3	B3/B4		-	1	Castrol_#3
-	-		C3/C4	1	Elf_#2
A3	B3/B4		C2/C3	1	Mobil_#2
**API Rating**				**Number of Oils**	**Oil Name(s)**
S *		C **			
SN		CF		11	Castrol_#4, Castrol_#5, Motul_#1, Elf_#1, Millers_#1, Millers_#2, Millers_#3, Mannol_#2, Lotos, Shell, Total_#3
SN		-		9	Castrol_#1, Castrol_#2, Castrol_#6, Mannol_#1, Fuchs_#2, Loqui, Ravenol, Motul_#2, Agip
SN		CF/CE		1	Wolver
SN/SM		CF		3	Eneos_#2, Eneos_#4, Revline
SM		CF		1	Total_#1
SM/SF		-		1	Xado_#1
SM/SL		CF		1	Mobil_#2
SM/SL		-		1	Mobil_#1
SL		-		1	Mobil_#3
SL		CF		3	Castrol_#3, Total_#2, Elf_#3
Not specified				9	Eneos_#1, Eneos_#3, Elf_#2, Valvoline, Fuchs_#1, Millers_#4, Gulf, Xado_#2, Orlen

* S (Service)—for petrol engines (positive-ignition/PI), ** C (Commercial)—for diesel engines (compression ignition/CI). (A)—oil for petrol engines; (B)—oil for light-duty diesel engines (passenger cars and light-duty commercial vehicles); (C)—oil for engines equipped with modern filtering and exhaust control (catalytic) systems.

**Table 3:** Assignment of the oils into the different groupings according to similarity in impedance magnitude values.

Group No. 1	Group No. 2	Group No. 3	Group No. 4	Group No. 5
Castrol_#3 Castrol_#4 Eneos_#2 Gulf Mannol_#1 Millers_#2 Ravenol Revline Total_#1 Xado_#1	Castrol_#1 Castrol_#6 Elf_#2 Eneos_#3 Eneos_#4	Agip Castrol_#2 Castrol_#5 Elf_#1 Eneos_#1 Loqui Millers_#1 Mobil_#1 Mobil_#2 Motul_#1 Motul_#2 Orlen Shell Total_#3 Valvoline Wolver Xado_#2	Fuchs_#1 Fuchs_#2 Millers_#3 Millers_#4	Elf_#3 Lotos Mannol_#2 Mobil_#3 Total_#2

**Table 4:** Assignment of the oils into different groupings according to the similarity in phase shift values.

Group No. 1	Group No. 2	Group No. 3	Group No. 4	Group No. 5
Elf_#3 Lotos Mannol_#2 Mobil_#3 Total_#2	Castrol_#3 Eneos_#2 Eneos_#3 Eneos_#4 Mannol_#1 Millers_#3 Millers_#4 Revline Total_#1	Agip Elf_#1 Eneos_#1 Fuchs_#1 Fuchs_#2 Gulf Loqui Millers_#1 Motul_#1 Motul_#2 Orlen Total_#3 Valvoline Wolver Xado_#1 Xado_#2	Castrol_#2 Castrol_#5 Castrol_#6 Elf_#2 Millers_#2 Mobil_#1 Mobil_#2 Ravenol Shell	Castrol_#1 Castrol_#4

**Table 5:** Assignment of the oils into the different groupings according to similarity in *Q-factor* (quality factor) values.

Group No. 1	Group No. 2	Group No. 3	Group No. 4	Group No. 5
Castrol_#1 Castrol_#4 Castrol_#5 Castrol_#6 Elf_#1 Millers_#2 Mobil_#1 Ravenol Total_#3 Xado_#1 Xado_#2	Castrol_#3 Elf_#3 Eneos_#2 Eneos_#3 Lotos Mannol_#2 Mobil_#3 Revline Total_#2	Fuchs_#1 Fuchs_#2 Motul_#1 Orlen Valvoline	Agip Castrol_#2 Elf_#2 Eneos_#1 Gulf Loqui Millers_#1 Mobil_#2 Motul_#2 Shell Wolver	Eneos_#4 Mannol_#1 Millers_#3 Millers_#4 Total_#1

**Table 6:** Assignment of the oils into the different groupings according to similarity in conductance values.

Group No. 1	Group No. 2	Group No. 3	Group No. 4	Group No. 5
Elf_#3 Eneos_#4 Lotos Mannol_#2 Mobil_#3 Total_#2	Castrol_#3 Eneos_#2 Eneos_#3 Mannol_#1 Millers_#3 Revline Total_#1	Castrol_#1 Elf_#2	Agip Castrol_#2 Castrol_#4 Castrol_#5 Castrol_#6 Elf_#1 Eneos_#1 Gulf Millers_#1 Millers_#2 Mobil_#1 Mobil_#2 Motul_#2 Ravenol Shell Total_#3 Valvoline Wolver Xado_#1 Xado_#2	Fuchs_#1 Fuchs_#2 Loqui Millers_#4 Motul_#1 Orlen

**Table 7:** Assignment of the oils into the different groupings according to similarity in susceptance values.

Group No. 1	Group No. 2	Group No. 3	Group No. 4	Group No. 5
Elf_#3 Mannol_#2 Mobil_#3 Total_#2	Agip Castrol_#2 Castrol_#5 Elf_#1 Eneos_#1 Loqui Millers_#1 Mobil_#1 Mobil_#2 Motul_#1 Orlen Total_#1 Total_#3 Valvoline Wolver Xado_#2	Castrol_#3 Castrol_#4 Eneos_#2 Gulf Lotos Mannol_#1 Millers_#2 Motul_#2 Ravenol Revline Shell Xado_#1	Fuchs_#1 Fuchs_#2 Millers_#3 Millers_#4	Castrol_#1 Castrol_#6 Elf_#2 Eneos_#3 Eneos_#4

**Table 8:** The assignment of the oils into the different groupings according to similarity in capacitance values.

Group No. 1	Group No. 2	Group No. 3	Group No. 4	Group No. 5
Agip Eneos_#1 Loqui Mannol_#2 Motul_#1 Orlen	Fuchs_#1 Fuchs_#2 Millers_#3 Millers_#4	Castrol_#2 Castrol_#3 Castrol_#5 Elf_#1 Elf_#3 Mannol_#1 Millers_#1 Mobil_#1 Mobil_#2 Mobil_#3 Motul_#2 Revline Shell Total_#1 Total_#2 Total_#3 Valvoline Wolver Xado_#1 Xado_#2	Castrol_#4 Eneos_#2 Gulf Lotos Millers_#2 Ravenol	Castrol_#1 Castrol_#6 Elf_#2 Eneos_#3 Eneos_#4

## Data Availability

Onedrive.
